# Manual Characterization of Sleep Spindle Index in Patients with Narcolepsy and Idiopathic Hypersomnia

**DOI:** 10.1155/2014/271802

**Published:** 2014-04-01

**Authors:** Lourdes M. DelRosso, Andrew L. Chesson, Romy Hoque

**Affiliations:** Division of Sleep Medicine, Department of Neurology, Louisiana State University School of Medicine, Shreveport, LA 71103, USA

## Abstract

This is a retrospective review of PSG data from 8 narcolepsy patients and 8 idiopathic hypersomnia (IH) patients, evaluating electrophysiologic differences between these two central hypersomnias. Spindles were identified according to the AASM Manual for the Scoring of Sleep and Associated Events; and counted per epoch in the first 50 epochs of N2 sleep and the last 50 epochs of N2 sleep in each patient's PSG. Spindle count data (mean ± standard deviation) per 30 second-epoch (spindle index) in the 8 narcolepsy patients was as follows: 0.37 ± 0.73 for the first 50 epochs of N2; 0.65 ± 1.09 for the last 50 epochs of N2; and 0.51 ± 0.93 for all 100 epochs of N2. Spindle index data in the 8 IH patients was as follows: 2.31 ± 2.23 for the first 50 epochs of N2; 2.84 ± 2.43 for the last 50 epochs of N2; and 2.57 ± 2.35 for all 100 epochs of N2. Intergroup differences in spindle count in the first 50 N2 epochs, the last 50 N2 epochs, and all 100 epochs of scored N2 were significant (*P* < 0.01) as were the intragroup differences between the first 50 N2 epochs and the last 50 N2 epochs.

## 1. Introduction

The American Academy of Sleep Medicine Manual for the Scoring of Sleep and Associated Events (Scoring Manual) defines a sleep spindle as a train of waves with frequency of 11–16 Hz, duration of more than 0.5 seconds, and maximal amplitude usually in the central derivations. Sleep spindles constitute an important marker for staging N2 sleep [1]. Sleep spindles, generated in the thalamus, are thought to raise the arousal threshold and prevent external stimuli signals from being transmitted to the cortex [2]. Earlier research has shown a statistically significant difference in the sleep spindle density in patients with N and IH when compared to controls and concluded that sleep spindle density could be a marker of a weakened mechanism of awakening [3]. The goal of our present study is to assess the spindle density in full night PSG using Scoring Manual criteria to evaluate electrophysiologic differences between narcolepsy and idiopathic hypersomnia.

## 2. Methods

### 2.1. Selection Criteria

Over a 2-year period (03.01.2010 to 03.10.2012), all patients with a new diagnosis of N or IH were identified from patient records at the Sleep Disorders Center of the Louisiana State University Health Sciences Center in Shreveport, Louisiana. The diagnosis of N and IH was made by a board certified sleep medicine physician based on history, clinical symptoms, and nocturnal PSG and MSLT according to ICSD-2 criteria.

To these N and IH patient records we applied the following exclusion criteria: presence of obstructive sleep apnea defined by total sleep time (TST) apnea hypopnea index >5; circadian disorder; insufficient sleep syndrome based on sleep diaries; positive drug screen performed with MSLT; REM sleep altering/cataplexy suppressing medications (e.g., tricyclic antidepressants, selective serotonin and/or norepinephrine reuptake inhibitors, and sodium oxybate), spindle altering medications (benzodiazepines); PSG/MSLT done at another facility; psychiatric or neurological comorbidity; and PSG/MSLT recording obscured by significant artifact. The remaining patients for the study consisted of 8 patients with N and 8 patients with IH. The PSG and MSLT studies used to establish a hypersomnia related diagnosis and used for this study data for all 16 patients were performed before stimulant initiation and off any other sleep/wake altering medications for >2 weeks.

### 2.2. Data Acquisition

All patients had a nocturnal PSG followed by an MSLT. The PSG recordings were acquired using the Alice 5 system (Respironics, Inc., Murrysville, PA, USA) and included 6 electroencephalogram channels (F3/A2, F4/A1, C3/A2, C4/A1, O1/A2, and O2/A1); vertical and horizontal electroculograms; chin and bilateral tibialis anterior muscle electromyogram; 1-channel electrocardiogram; and respiratory monitors (nasal pressure transducer, thermistor, thoracic and abdominal plethysmography belts, microphone, and pulse oximetry). PSG scoring was in accordance with the Scoring Manual.

### 2.3. Quantification of Spindles

Each PSG was scored in agreement by 2 board certified sleep medicine physicians (LD, RH) in accordance with the Scoring Manual. Individual spindle was counted per 30-second epoch for the first 50 epochs of stage N2 sleep and the final 50 epochs of stage N2 sleep. Spindles were defined, as per the Scoring Manual, as a train of distinct waves with frequency of 11–16 Hz with duration of ≥0.5 seconds, usually maximal in the central derivations.

### 2.4. Calculation of Spindle Index

Spindle index, the total number of individual spindles per epoch of stage N2 sleep, was calculated for the first 50 epochs of stage N2 sleep and the last 50 epochs of stage N2 sleep.

### 2.5. Statistical Analysis

Assessment of statistical significance for samples was performed using the nonparametric Mann-Whitney *U* calculation. *P* values less than 0.05 were considered statistically significant.

## 3. Results

### 3.1. Demographics

Demographic data for the two groups is presented in Table 1. Mean age for the N group was 27.5 years (range: 11–33); 4 patients were women. Mean age for the IH group was 33.12 years (range: 20–57); 7 patients were women. Age, body mass index, and Epworth Sleepiness Scale Score were not significantly different between the N and IH groups. Four patients with N exhibited cataplexy and four did not.

### 3.2. PSG Data

**Figure 1:**
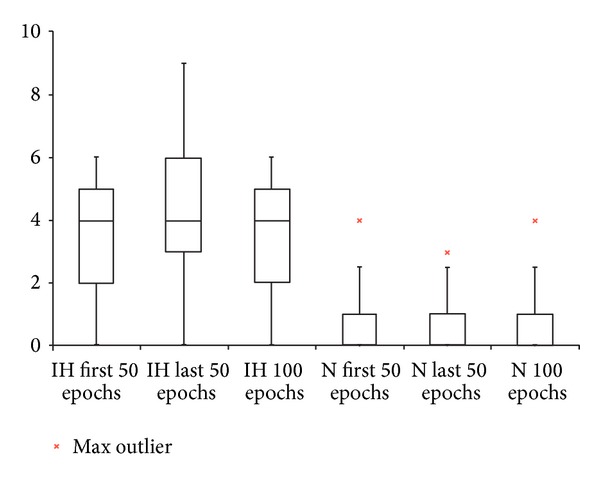
Box-plot comparison of sleep spindle index in idiopathic hypersomnia (IH) and narcolepsy (N). Max: maximum.

PSG data is summarized in Table 2(a). Mann-Whitney *U* calculations did not show significant differences between the two groups in total sleep time, wake after sleep onset, sleep efficiency, and sleep stage percentages. Intergroup differences in spindle count in the first 50 epochs of N2, the last 50 epochs of N2, and all 100 epochs of scored N2 were statistically significant (*P* < 0.01) with IH having a higher N2 sleep spindle index compared to N. While box-plot comparison shows overlap between the sleep spindle index values for IH and N, the sleep spindle index values for IH are consistently higher than those in N (Figure 1). Intragroup differences between the first 50 N2 epochs and the last 50 N2 epochs were also significant (*P* < 0.01) with higher N2 sleep spindle densities in the final 50 epochs in both conditions (Table 2(b)).

The first 50 epochs and last 50 epochs of N2 sleep were often not continuous and interrupted by other sleep stages in both patients with IH and N (Tables 3(a) and 3(b)). The number of continuous N2 periods was similar between both IH and N, with no statistically significant differences noted between N and IH. The only statistically significant difference in the number of continuous N2 periods was between the number of N2 periods evaluated in the first 50 epochs of N2 sleep and the last 50 epochs of N2 sleep in IH.

## 4. Discussion

Our study demonstrates a statistically significant difference in sleep spindles between patients diagnosed with IH and patients with N and a statistically significant increase in sleep spindle density in the last 50 epochs of N2 compared to the first 50 epochs of N2 of both conditions.

The increased spindle density noted in the last 50 epochs of N2 sleep in both IH and N is a finding that will require further study. In the patients with IH, N2 fragmentation was significantly less in the last 50 epochs of N2 compared to the first 50 epochs of N2. This was not the case for N, making it unlikely that sleep fragmentation played a key role in spindle density increases noted in the last 50 epochs of both IH and N.

The difference in spindle density between IH and N may be in part due to different pathophysiology in these conditions. N, with or without cataplexy, is characterized by rapid eye movement instability, nocturnal sleep disruption, and daytime sleepiness. The putative pathophysiological mechanism is loss of hypothalamic hypocretin neurons [4]. IH presents in different clinical forms, but, typically, patients report difficulty waking with frequent returns to sleep and prolonged unrefreshing naps. Hypothalamic dysfunction is not suspected in these patients [5]. The thalamus has crucial roles both in maintenance of wakefulness and promotion of NREM sleep and is essential in the production of spindles [6, 7]. Spindles have been shown to protect sleep from arousal stimuli [8]. The increased spindle index found in IH may explain the symptoms of difficulty waking and “sleep drunkenness” [9]. A hypersomnia syndrome similar to idiopathic hypersomnia has been described in isolated cases of bilateral thalamic strokes. An increase in spindle density up to 100% has also been seen in these patients, with an increased spindle density over time [10, 11]. An increased spindle index may potentially serve as a marker of decreased cortical arousability.

Spindle density may be affected by patient's gender. In a small study of 5 men and 5 women, spindle density was higher in females compared to males [12]. The differences in spindle index in our two groups may be in part due to gender differences between the two groups; a larger study may help elucidate gender spindle index differences. Spindle density may also increase during the luteal phase; and menstrual hypersomnia, a recurrent hypersomnia, occurs during the luteal phase suggesting a potential association with spindle density [13, 14]. The menstrual cycle location was not assessed in our female patients prior to PSG.

The precise effect of age on spindle frequency is unclear. Prior research is inconsistent regarding the effect of age on spindle frequency when using automated spindle frequency quantification, as both increasing spindle frequency and decreasing spindle frequency have been reported with increasing age. Our study has the advantage of using nonautomated manual quantification [15, 16].

Limitations of our study include patients diagnosed in a single institution, small number of patients, single night PSG data for each patient, retrospective analysis, lack of blind comparison, and lack of age matched healthy controls. In addition, the variance in spindle index for both groups is large, making it difficult to determine a spindle index cut-off differentiating these two conditions.

Our study is suggestive of a difference in spindle index but requires further examination in a larger trial. Spindle index when used in combination with clinical history and other neurophysiologic markers may further substantiate a clinical distinction between N and IH.

## Figures and Tables

**Table 1:** Demographics.

Demographics data	8 patients with narcolepsy	8 patients with idiopathic hypersomnia	Mann-Whitney *P* values
Age: mean ± SD, (range)	27.5 ± 12.73, (11–55)	32.62 ± 14.34, (20–57)	0.674
ESS: mean ± SD, (range)	19.75 ± 2.92, (16–24)	16.63 ± 3.62, (10–20)	0.074
BMI: mean ± SD, (range)	33.69 ± 7.37, (24–43.8)	28.69 ± 5.35, (20.9–37.8)	0.156
Sex (F): total, % of total	4, 50%	7, 87%	Not applicable

BMI: body mass index; ESS: Epworth Sleepiness Scale Score; SD: standard deviation.

**Table:** (a)

PSG variable	8 patients with narcolepsy Mean ± SD	8 patients with idiopathic hypersomnia Mean ± SD	Mann-Whitney *P* values
Total sleep time	419.56 ± 47.96	461.31 ± 33.35	0.036
Wake after sleep onset	37.94 ± 23.41	32.5 ± 31.91	0.372
Sleep efficiency	90.89 ± 5.08	92.49 ± 5.99	0.674
N1 %	3.36 ± 3.41	3.06 ± 1.9	0.793
N2 %	55.5 ± 14	61.2 ± 10.65	0.345
N3 %	16.38 ± 12.5	17.83 ± 4.04	0.294
REM sleep %	24.76 ± 6.43	17.91 ± 8	0.027*
Sleep spindle index: 1st 50 epochs of N2	0.37 ± 0.73	2.31 ± 2.23	0.001*
Sleep spindle index: last 50 epochs of N2	0.65 ± 1.09	2.84 ± 2.43	0.001*
Sleep spindle index: 100 epochs of N2	0.51 ± 0.93	2.57 ± 2.35	0.001*

PSG: polysomnogram; REM: rapid eye movement sleep; SD: standard deviation. *P value less than 0.05.

**Table:** (b)

Comparison	Mann-Whitney *P* values
N: 1st 50 epochs N: last 50 epochs	0.006

IH: 1st 50 epochs IH: last 50 epochs	0.001

N: narcolepsy group; IH: idiopathic hypersomnia group.

**Table:** (a)

	Mean ± SD	Range
Idiopathic hypersomnia		
1st 50 epochs	3.37 ± 1.06	2–5
Last 50 epochs	1.37 ± 0.51	1-2
Narcolepsy		
1st 50 epochs	2.87 ± 0.99	1–4
Last 50 epochs	2.0 ± 1.30	1–4

**Table:** (b)

Comparison	Mann-Whitney *P* values
IH: 1st 50 epochs IH: last 50 epochs	0.002

N: 1st 50 epochs N: last 50 epochs	0.207

IH: all epochs N: all epochs	0.850

IH: 1st 50 epochs N: 1st 50 epochs	0.400

IH: last 50 epochs N: last 50 epochs	0.462

IH: idiopathic hypersomnia; N: narcolepsy.
